# An Overview of 10^th^ Anniversary of *Cells*—Advances in Cell Nuclei: Function, Transport and Receptors

**DOI:** 10.3390/cells12010055

**Published:** 2022-12-23

**Authors:** Hiroshi Miyamoto

**Affiliations:** 1 Department of Pathology & Laboratory Medicine, University of Rochester Medical Center, Rochester, NY 14642, USA; hiroshi_miyamoto@urmc.rochester.edu; Tel.: +1-585-275-8748; 2 Department of Urology, University of Rochester Medical Center, Rochester, NY 14642, USA; 3 James P. Wilmot Cancer Institute, University of Rochester Medical Center, Rochester, NY 14642, USA

The year 2021 marked the 10^th^ anniversary of the publication of *Cells*. In this year, the journal published more than 4000 papers, and the journal website attracted more than 50,000 monthly page views. We were also delighted and proud to have published a series of Special Issues and hosted a variety of events. To celebrate this important milestone, a Special Issue entitled, “10^th^ Anniversary of *Cells*—Advances in Cell Nuclei: Function, Transport and Receptors,” was launched in our section. This Special Issue, composed of six original research articles [1,2,3,4,5,6] and six reviews [7,8,9,10,11,12], covers various aspects of the function of cell nuclei, transport and/or receptors.

For each cell to function properly, the necessary proteins must be synthesized from the genetic information flow, which is dependent on the transcription and degradation rates of mRNAs, as well as on the translation and degradation rates of proteins. The authors’ group previously compared omics data to study the stability of mRNAs and proteins, and their synthesis/degradation rates in a yeast model [13]. Forés-Martos et al. [1] further determined if all these observations in *Saccharomyces cerevisiae* were seen in other organisms, including the yeast *Schizosaccharomyces pombe*, the human cervical cancer HeLa cell line and the bacterium *Escherichia coli*. An analysis of omics data revealed the similarities in gene expression fluxes among the four organisms. The new findings also support the hypothesis that the transcription rate in actively growing organisms is the main determinant of the amount of the corresponding protein. The authors further suggest that comparing the rates of transcription/translation/degradation and the stability of mRNAs and proteins in a series of organisms is helpful for elucidating how the environment of cells has an impact on gene expression regulation.

During apoptosis, the best known form of programmed cell death, various proteins are translocated from or to the nucleus. The assessment of the subcellular localization of these proteins is, therefore, useful, particularly for investigating the functional activity of apoptosis regulators. For this purpose, adequate subcellular fractionation techniques that distinguish apoptotic bodies and cell fragments from their contaminants are of importance. Senichkin et al. [2] compared multiple nucleus/cytoplasm fractionation methods, including those using a Potter–Elvehjem homogenizer, non-ionic detergents (i.e., digitonin and NP-40) and the stepwise lysis of cells and washing of the resultant nuclei with NP-40 at both steps. The authors eventually developed a simple and efficient protocol to reliably fractionate the nucleus and cytoplasm of both viable and apoptotic cells.

Aberrant RNA splicing is thought to contribute to the development of various types of malignancies, including breast cancer. Indeed, the alternative splicing of several genes, such as *serine/arginine-rich splicing factors* (*SRSFs*), has been implicated in not only breast carcinogenesis, but also disease progression and poorer prognosis [14]. Oh et al. [3] performed next-generation sequencing in two human breast cancer cell lines, representing those with low vs. high metastatic potential. After validation of the results in two additional cell lines, the enrichment of RNA-binding motifs recognized by SRSFs was explored. Then, the alternate splicing of a SRSF1-regutad target, *DCUN1D5*, with the skipping of exon 4 in the pre-mRNA to introduce a premature termination codon was suggested to be closely associated with breast cancer progression. In the TCGA dataset of breast cancer, the elevated expression of *DCUN1D5* was also found to be associated with significantly poorer patient outcomes.

In the era of Prostate Imaging Reporting and Data System (PI-RADS scoring, a combination of a systematic biopsy of the prostate and a magnetic resonance imaging-targeted biopsy is expected to yield much higher sensitivity in the detection of cancer lesions [15]. However, the PI-RADS score may not always precisely predict the presence of clinically significant prostate cancer. Keck et al. [4] measured the serum levels of seven microRNAs in men undergoing targeted prostate biopsy. Of those examined, miR-486 and let-7c were found to considerably improve the prediction of cancer detection. Particularly in those with a PI-RADS 3 or higher lesion, the sensitivity for detecting Grade Group 2 or higher cancer and the negative predictive value for the absence of such a cancer were 95.2% and 97.1%, respectively. These findings indicate that the further combination with serum miR-486/let-7c testing more accurately detects clinically significant prostate cancer.

Chemotherapy with taxanes, such as docetaxel, has often been used in men with castration-resistant prostate cancer. Although significant survival benefits of taxane therapy have been documented, these patients inevitably encounter drug resistance, for which the underlying molecular mechanisms are far from being fully understood [16]. Ortiz-Hernandez et al. [5] determined the role of LEDGF/p75 containing an integrase-binding domain (IBD), as well as its interacting partners, in chemoresistance in docetaxel-resistant prostate cancer cells where the expression of these proteins was upregulated. Co-immunoprecipitation showed the co-localization of LEDGF/p75 with IBD-interacting proteins, including JPO2, menin and HRP2. In addition, the knockdown of each in docetaxel-resistant prostate cancer cells resulted in the enhancement of its cytotoxicity while increasing apoptosis. These observations indicate that the LEDGF/p75 interactome plays an important role in the induction of docetaxel resistance in prostate cancer.

Chromosomal rearrangements affecting the nucleoporin *NUP98* gene have been implicated in the development of hematological malignancies. For instance, the fusion of NUP98 with a homeobox protein HOXA9, resulting in alterations in nuclear envelope organization, has been detected in leukemias [17]. Vaz et al. [6] further investigated the involvement of a tumor suppressor retinoblastoma protein (RB) in NUP98-HOXA9 fusion protein-mediated changes in the nuclear envelope. The aberrant nuclear envelope was found not to occur in cells lacking RB. Furthermore, RB loss reduced the associations of NUP98-HOXA9 with H3K4me3 or H3K27me3, an epigenetic modification to the DNA packaging protein histone H3. Thus, RB could be a key regulator of NUP98-HOXA9 fusion and might, thereby, contribute to leukemogenesis.

Yadav et al. [7] reviewed various characteristics of coronavirus disease 2019 (COVID-19), such as the genomic arrangement, structural organization/assembly, nonstructural proteins, accessory factors and life cycle, as well as potential molecular targets for drug design. Merighi et al. [8] summarized the role of purinergic signaling mediated by adenosine or ATP receptors in the development of Alzheimer’s disease and discussed related potential therapeutic strategies. Abaandou et al. [9] comprehensively covered the achievements, such as genetic engineering approaches and functional genomic and bioinformatic analyses, in a human embryonic kidney cell line HEK293 and its derived sublines, which have been being used in a wide variety of research. Soroka et al. [10] highlighted the utility of the loop-mediated amplification of DNA, especially to address if this growing technique is superior to polymerase chain reactions in, for instance, detecting COVID-19. Kachaev et al. [11] outlined the nuclear localization and functional role of the translation apparatus, including translation factors and ribosomal proteins, in the nucleus of eukaryotic cells. Hamed and Antonin [12] digested how nucleoporins could interact with the nuclear pore membrane and, subsequently, contribute to the assembly and function of nuclear pore complexes that regulate the transport of macromolecules between the nucleus and cytoplasm. 

In conclusion, a variety of aspects of cell nuclei research are described in this Special Issue. These observations provide unique insights into the function of cell nuclei, transport and receptors.
